# "With a Little Help from My Friends"—Social Motility in *Trypanosoma brucei*

**DOI:** 10.1371/journal.ppat.1005272

**Published:** 2015-12-17

**Authors:** Edwin A. Saada, Stephanie F. DeMarco, Michelle M. Shimogawa, Kent L. Hill

**Affiliations:** 1 Department of Microbiology, Immunology and Molecular Genetics, University of California, Los Angeles, Los Angeles, California, United States of America; 2 Molecular Biology Institute, University of California, Los Angeles, Los Angeles, California, United States of America; University of Wisconsin Medical School, UNITED STATES

## Microbial Social Behavior: “The Whole Is Greater Than the Sum of Its Parts”

In their natural environments, microbes are not found in isolation, but live in groups where the ability to communicate and cooperate with friends, while thwarting activities of enemies, is of paramount importance [1,2]. The capacity for interaction among cells in a group allows for social behaviors, which present as emergent properties of the group as a whole that are not predicted from the sum of activities of individual cells. One example includes quorum sensing (QS), which enables cells in a population to coordinate gene expression and limit premature expenditure of resources. Other social behaviors include those occurring in the context of surfaces, such as biofilm formation and various forms of swarming motility across surfaces, as seen in bacteria and slime molds [3–5].

**Fig 1:**
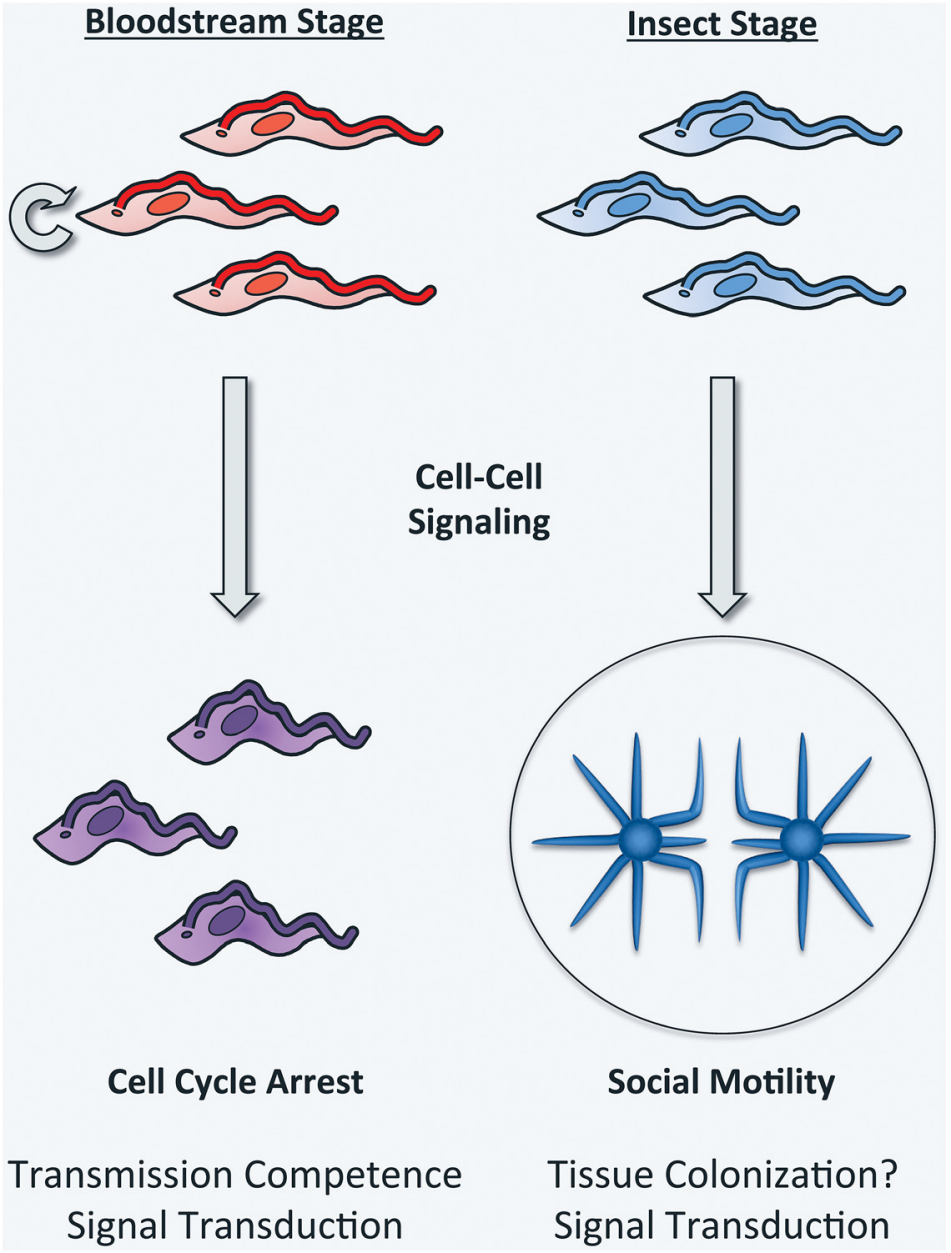
Trypanosomes are social. Trypanosome cell–cell interactions operate in bloodstream and insect stage parasites. In the bloodstream, “long slender form” parasites (red) differentiate into growth-arrested “short stumpy forms” (purple) through a quorum sensing-mediated mechanism. Stumpy parasites are pre-adapted for the tsetse fly environment and the transition thus establishes transmission competence, while also limiting bloodstream parasitemia [52,53]. Procyclic *T*. *brucei* (insect midgut stage, blue) undergo social motility when cultivated on semi-solid surfaces, using cell–cell signaling to promote collective motility across the surface and coordinating their movements in response to extracellular signals from nearby parasites. This leads to formation of radial projections that extend outward from the initial site of inoculation [8]. These activities are hypothesized to support colonization and/or transit of tissue surfaces in the fly. Beyond their direct impact on understanding parasite development, recent studies of stumpy formation and social motility have provided insight into parasite signal transduction. See text for details.

Recognizing cell–cell communication and social behaviors as ubiquitous among bacteria transformed our views of microbiology and microbial pathogenesis [1,2]. Parasitic protozoa cause tremendous human suffering worldwide and, although they engage in cell–cell interactions, the paradigm of social behavior is not commonly applied in studies of these organisms. One long-known social activity in protozoan parasites is QS-driven differentiation of the African trypanosome, *Trypanosoma brucei*, in the bloodstream of a mammalian host. During infection, proliferating *T*. *brucei* cells undergo cell-density dependent differentiation into quiescent forms that are then competent to be transmitted by a tsetse fly vector (Fig 1) [6,7]. More recently, the discovery of social motility (SoMo) in insect-stage *T*. *brucei* (Fig 1) [8] has highlighted the capacity of these organisms for group-level behavior, and several recent studies of SoMo emphasize the potential for concepts underlying social behavior to provide insight into parasite biology.

## Discovery of Social Motility in *Trypanosoma brucei*

African trypanosomes cause sleeping sickness in humans and nagana in livestock. They impose a substantial medical and economic burden across nearly 30 countries of sub-Saharan Africa, where approximately 70 million people live at risk of infection [9–11]. The parasites are transmitted between mammalian hosts by blood-feeding tsetse flies. Most studies and animal infection models consider *T*. *brucei* as individual cells in suspension, yet in their natural environments these parasites frequently live in contact with tissue surfaces. This is most evident in the tsetse fly, where trypanosomes undergo extensive movements across fly tissues, culminating in colonization of the salivary gland epithelium and subsequent differentiation into human-infectious forms [12].

To investigate the influence of surface interactions on trypanosome biology, Oberholzer and Lopez et al. cultivated procyclic form (insect midgut stage) *T*. *brucei* on semisolid agarose [8]. They discovered that parasites assemble into groups of cells that undergo collective movements, forming multicellular projections that radiate outward from the site of inoculation. Although individual parasites can move freely within a group, movement of the group is polarized such that it advances at a single, leading edge. Moreover, groups alter their movements when they sense other parasites nearby. The combined data reveal that procyclic trypanosomes can sense extracellular signals and coordinate their activities in response to these signals. This behavior was termed “social motility” based on analogies to social motility and other surface-associated motilities in bacteria.

## Why Be Social?

In bacteria, social behaviors provide numerous advantages, including enhanced ability to colonize, transit, and penetrate surfaces; enhanced protection from host defenses; improved accessibility to nutrients; and opportunities for genetic exchange [1,2,5,13,14]. Although in vivo manifestations and ramifications of *T*. *brucei* social motility are not yet known, the parasites can foreseeably benefit from the same advantages afforded to bacteria.

To complete their transmission through the tsetse fly, trypanosomes must undertake an epic journey fraught with hazards [15–17]. Under optimized laboratory conditions, less than 20% of fly infections yield mammalian-infectious parasites, and rates in the field are even lower [17,18]. The journey begins when ingested bloodstream parasites differentiate into procyclic-stage parasites that colonize the midgut lumen and must then get through or around the peritrophic matrix (PM) to establish infection on the midgut epithelium. The PM is a chitinous lining that protects the gut epithelium from digestive enzymes and presents a formidable barrier to *T*. *brucei* infection [19,20]. Once beyond the PM, parasites must penetrate the proventriculus and move along the alimentary tract to the mouth parts. From there, they move up the salivary duct to the salivary gland, where they colonize the gland epithelium and complete differentiation into human-infectious forms. Along the way, *T*. *brucei* must compete for resources while overcoming harsh conditions and fly defenses, including antimicrobial peptides and lectins [16,21].

Extensive surface colonization and the arduous nature of parasite movement through the fly present bottlenecks to infection [16,22] and led Oberholzer and Lopez et al. to propose that SoMo supports one or more of these in vivo activities [8]. Recent work from the Roditi group has strengthened this hypothesis [23,24]. Prior work defined parasites appearing within the first few days of tsetse infection as “early” procyclics, while “late” procyclics are those that appear at later time points [25], after parasites have persistently colonized the ectoperitrophic space. Early and late procyclics can be distinguished by differences in surface protein expression [23]. Imhof and colleagues discovered that SoMo initiation is cell density-dependent and that early procyclics cultured in vitro undergo social motility, while late procyclics do not. Importantly, they showed that SoMo competence is linked to the early developmental stage, rather than to the presence of specific surface proteins that distinguish between early and late developmental stages. The precise point at which the switch from early to late procyclics occurs is not yet clear but is hypothesized to correlate with infiltration of the ectoperitrophic space, suggesting that SoMo may be a property of cells that colonize and penetrate the peritrophic matrix. Further evidence linking SoMo to successful midgut infection comes from studies of a null mutant of the Requires Fifty Three (*RFT1)* gene, which is required for N-glycosylation of parasite proteins [26]. *RFT1* null mutants show defective SoMo in vitro and exhibit reduced and delayed establishment of midgut infections in the fly [24]. Thus, these combined studies establish SoMo as a property of a specific parasite developmental stage and correlate the in vitro behavior to a critical step of the in vivo transmission cycle.

## More Than an Oar: The Trypanosome Flagellum Contains cAMP Signaling Systems That Control Social Motility

A universal requirement of microbial social behavior is the ability of cells to sense and respond to extracellular signals, and SoMo therefore provides a novel opportunity for dissecting trypanosome signal transduction. This presents an important advance because perception and transduction of extracellular signals are important features of parasite biology [27], but underlying mechanisms are poorly understood. In addition to a well-known role in motility, the conserved role of the eukaryotic flagellum (also known as cilium) as a signaling center has emerged as a unifying theme in vertebrate development and pathophysiology of human genetic diseases [28]. This concept, together with the prominent role of cyclic nucleotides in cilium-dependent signaling and in control of microbial social behaviors [29–32], made flagellar cyclic-AMP (cAMP) a focal point for interrogation of SoMo signaling mechanisms.

Cellular cAMP levels are controlled through opposing activities of adenylate cyclases (ACs) and cAMP-specific phosphodiesterases (PDEs). Through proteomic analysis of flagellar membranes, Saada et al. identified *T*. *brucei* ACs that are localized to specific flagellum subdomains and upregulated in the procyclic life cycle stage [33], making them good candidates to test for a role in SoMo. Utilizing gene-specific RNAi, Lopez et al. found that knockdown of certain procyclic-specific ACs enhanced social motility, while knockdown of others had no effect [34]. The findings reveal that individual AC isoforms have specialized functions and indicate that a subset of ACs coordinates the response to signals governing SoMo. Importantly, AC point mutants that disrupt catalytic activity phenocopy RNAi knockdown, supporting the idea that attenuating cAMP production, as opposed to total loss of the protein, activates SoMo.

In complementary studies, Oberholzer and colleagues found that pharmacological or genetic inhibition of cAMP-specific phosphodiesterase B1 (PDEB1) blocks SoMo, indicating that elevated cAMP levels are inhibitory [35]. Supporting this, cAMP levels in live trypanosomes were monitored using a FRET sensor, demonstrating that SoMo inhibition directly correlates with a rise in intracellular cAMP. Additionally, membrane-permeant cAMP or non-hydrolyzable cAMP analogues were found to inhibit SoMo. Surprisingly, the authors also found that the SoMo defect of PDEB1 knockdown cells is rescued when these mutants are co-cultured with wild type (WT) cells. To do this, they employed WT and PDEB1 knockdown cell lines, each expressing a different fluorescent protein, making it possible to independently monitor movement of WT and PDEB1 knockdown cells in a mixed culture. Despite being incapable of forming projections on their own, PDEB1 knockdown cells formed projections when mixed with WT cells. The result is the first demonstration of *trans*-complementation in trypanosomes and suggests that PDEB1 knockdown cells are competent for SoMo, but fail to engage in SoMo because they lack a factor that can be provided by WT cells.

Notably, PDEB1 localizes exclusively to the flagellum [36], as do all ACs analyzed thus far, with at least one AC involved in SoMo that is further restricted to the flagellum tip [33,34,37]. The combined data thus reveal that the *T*. *brucei* flagellum is specialized for cAMP signaling and suggest a model in which localized changes in cAMP levels within specific flagellum subdomains control SoMo [33–35,38]. In this model, PDEB1 acts as a diffusion barrier along the length of the flagellum to confine cAMP signaling output to the site of its production by specific ACs. In the absence of PDEB1, cAMP becomes elevated and diffuses throughout the flagellum, inhibiting activities that would otherwise drive SoMo [35]. Interestingly, regulation of SoMo by cAMP in *T*. *brucei* has parallels to cyclic-nucleotide regulation of swarming motility in bacterial pathogens. In *Pseudomonas* spp., for example, loss of a diguanylate cyclase that produces cyclic-di-GMP results in hyper-swarming behavior, whereas loss of the cyclic-di-GMP phosphodiesterase results in the inability to swarm at all [39,40].

Trypanosomal ACs have attracted interest because their domain structure suggests they may act as receptors for extracellular ligands [41]. However, the unusually large size of the AC gene family in *T*. *brucei* (>65 genes) and lack of assays for cAMP responses have hindered studies of these intriguing proteins. Likewise, although PDEs have been a focus of attention as a drug target in bloodstream-stage *T*. *brucei*, their function in fly life cycle stages has received little attention. Indeed, although important advances have been made recently [34,35,42–44], cAMP signaling in general remains poorly understood in trypanosomes. In this context, SoMo signaling studies have broad impact because, in addition to identifying genes governing social behavior, they advance understanding of cAMP signaling systems in trypanosomes and demonstrate that the concept of the flagellum as a signaling organelle extends to a group of important human and animal pathogens.

## Summary and Outlook

Studies of social motility in African trypanosomes have revealed new conceptual frameworks and produced novel approaches for considering these pathogens. For example, it was not previously possible to discern phenotypic differences between early and late procyclic developmental stages beyond the presence of a single surface protein. SoMo has been instrumental in establishing these as specific life cycle stages and defining a distinct developmental transition [23]. Likewise, although functional analyses of receptor-type adenylate cyclases previously provided insight into host-parasite interaction [44], such studies have been limited to a few members of this large and enigmatic protein family. SoMo now provides ready avenues for structure-function analyses of these understudied proteins [34,45]. More broadly, the ease of visualizing individual trypanosomes in a mixed community and the genetic tractability of *T*. *brucei* mean that SoMo can be exploited for elucidating principles of self-assembly and cell–cell interactions in microbial systems.

**Fig 2:**
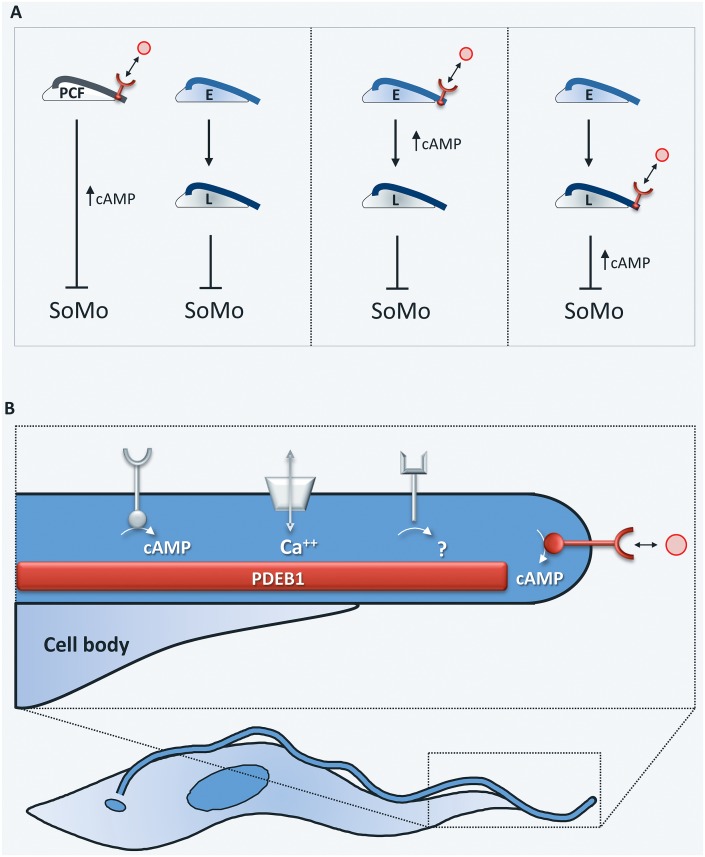
Regulation of social motility. **(A)** Three alternate models for regulation of social motility. (Left) Elevated cAMP at the flagellum tip (red) in response to regulation of tip-localized adenylate cyclase and the transition from early to late procyclics independently inhibit SoMo. (Middle) Elevated cAMP at the flagellum tip triggers the transition from early to late procyclics, which then inhibits SoMo. (Right) Development into late procyclics triggers elevated cAMP at the flagellum tip, which is then the inhibitory signal. **(B)** In addition to the known cAMP signaling systems that control SoMo (red), the trypanosome flagellum harbors several predicted signaling systems, e.g., ion transporters, kinases, additional ACs, and other receptor-like proteins [50,51] whose functions await discovery.

With foundations established, the stage is now set to tackle several key questions. An obvious area of interest will be to evaluate the role of SoMo and underlying signaling events during fly infection. With mutants now available, it will be possible to address this directly, and these efforts would be aided by development of methodologies for visualizing parasites in live tsetse flies. With regard to signaling mechanisms, it will be informative to elucidate the relationship between elevated cAMP and the late procyclic developmental stage, both of which inhibit SoMo. At least three models can be envisioned to explain these results (Fig 2A). For example, cAMP and the late procyclic developmental stage might act independently. Alternatively, elevated cAMP at the flagellum tip might promote the early to late developmental transition, which then inhibits SoMo. However, the presence of GPEET procyclin, a marker of early procyclics, in PDEB1 knockdowns [35] argues against this model. Finally, development into late procyclics might promote elevation of cAMP at the flagellum tip, which would be the inhibitory trigger. Further studies are needed to distinguish between these models. Additional important topics include investigation of cellular signaling within early versus late-stage procyclic cells, determining the role of AC extracellular domains and location, as well as identification of AC ligands.

Beyond cAMP, SoMo undoubtedly depends on additional cell-derived as well as non-cell-derived elements. Of particular interest are factors that drive assembly of parasites into groups, those that promote outward movement of these groups, and those that govern repulsion of groups from one another. Also important will be to define factors responsible for *trans*-complementation. In bacteria, *trans*-complementation can be achieved through transfer of outer membrane proteins from one cell to another, and multiple signaling systems have been implicated in coordinating swarming [46–49]. The trypanosome flagellum houses several signaling systems with the capacity for metabotropic and ionotropic responses to external signals—including ion transporters, kinases, ACs, and other putative receptors (Fig 2B) [50,51]—whose functions await discovery.

In closing, studies of social motility and quorum sensing have provided insight into trypanosome developmental biology and signal transduction, illustrating the value of considering these parasites in the context of microbial social behavior concepts. It will be informative to apply this paradigm more broadly among protozoan pathogens.
